# Fruit and Fruit-Derived Products of Selected Sambucus Plants as a Source of Phytosterols and Triterpenoids

**DOI:** 10.3390/plants14101490

**Published:** 2025-05-16

**Authors:** Otgonbileg Onolbaatar, Soyol Dashbaldan, Cezary Pączkowski, Anna Szakiel

**Affiliations:** 1 Department of Plant Biochemistry, Institute of Biochemistry, Faculty of Biology, University of Warsaw, 1 Miecznikowa Street, 02-096 Warsaw, Polandc.paczkowski@uw.edu.pl (C.P.); 2 School of Food, Light Industry and Design, Mongolian University of Science and Technology, 8nd Khoroo, Baga Toiruu 34, Sukhbaatar District, Ulaanbaatar 14191, Mongolia; soyol_d@must.edu.mn

**Keywords:** black elder, elderberry, food preparations, fruit-derived products, phytosterols, red elder, *Sambucus nigra*, *Sambucus racemosa*, triterpenoids

## Abstract

Plant-derived foods have gained recognition for their health-promoting values, which are largely attributed to bioactive compounds such as phytosterols and triterpenoids. This study aimed to analyze the content of these compounds in the fruit of black elder (elderberry) *Sambucus nigra* L. and in commercially available food products, including jam, juice, syrup and wine. An additional objective was to compare the phytosterol and triterpenoid profiles of fruits and fruit cuticular waxes from wild and cultivated elderberry (cultivar Haschberg), ornamental elderberry (*S. nigra* f. *porphyrophylla* cultivar Black lace “Eva”), and red elderberry (*S. racemosa*). Qualitative and quantitative determinations were performed using gas chromatography coupled with mass spectrometry (GC-MS). This study provides a detailed characterization of triterpenoids in black and red elderberries, revealing a complex composition of oleanane-, 18-oleanane-, ursane-, lupane- and taraxastane-type compounds. Elderberry fruits were found to be rich sources of phytosterols (ranging from 0.54 mg/g d.w. in cultivated elderberry cv. Haschberg to 0.96 mg/g in ornamental elderberry) and triterpenoids (from 1.41 mg/g d.w. in *S. racemosa* to 13.81 mg/g in ornamental elderberry). Among the processed products, jam contained the highest concentration of these compounds (a total of 340 µg/g) and wine contained the lowest (0.87 µg/mL). Furthermore, the results suggest that certain features of the triterpenoid profile in *S. nigra* and *S. racemosa* may hold chemotaxonomic significance for the Sambucus genus.

## 1. Introduction

In recent years, plant-derived foods have gained popularity and are highly valued by consumers not only for their taste and nutritional benefits but also for potential health-promoting properties, which are attributed to their bioactive constituents. Plant-derived bioactive compounds exhibit numerous pharmacological activities that might help mitigate the pathological impact of various human diseases, including cardiovascular and neurodegenerative disorders, diabetes, and cancer [1]. Therefore, the health-promoting properties of bioactive components from fruits, vegetables, cereals, and other plant sources have garnered significant attention from researchers, consumers, and producers of functional foods and dietary supplements. One of the plant food sources gaining increasing interest is the fruit of the black elderberry (*Sambucus nigra* L. ssp. *nigra*), which is particularly rich in phenolic antioxidants (including anthocyanins) and triterpenoids, mainly ursolic and oleanolic acids [2,3].

Elderberry fruit has long been used as a remedy in traditional medicine by many indigenous cultures and is renowned for its health-promoting effects [4,5,6]. These include antioxidant, antiviral, anti-diabetic, anticancer, and immunostimulatory properties, leading to the prevention of cardiovascular, neurodegenerative, and inflammatory diseases [7,8,9]. In folk medicine, elderberry was primarily used to treat respiratory tract disorders, including the common cold, flu, influenza and other viral infections [10]. Contemporary research has shown that elderberry fruit extract can block influenza virus glycoproteins and increase the expression of interleukin-8 (IL-8), interleukin-6 (IL-6), and tumor necrosis factor (TNF) [2,8,11]. Recently, elderberry products have been used in the treatment of novel infections caused by coronaviruses, including severe acute respiratory syndrome (SARS), Middle East respiratory syndrome (MERS), and the coronavirus disease of 2019 (COVID-19) [11,12]. These effects are currently being evaluated in in vitro and in vivo studies, as well as preclinical and clinical trials.

Although elderberry fruits are mildly poisonous in their raw state due to the presence of cyanogenic glycosides and lectins, they become edible and tasty after cooking. They have a long culinary tradition in the preparation of home-made pies, jelly, jams, chutneys, punch, wine, and liqueurs [3,13]. In the past, raw elderberry plant material for culinary or medicinal uses was exclusively collected from wild plants. However, with the breeding of elderberry crop cultivars (including Alleso, Korsor, Samyl, and Sampo, some of the most popular varieties), elderberries are now cultivated commercially in many countries [3,14,15]. As a result, they have become widely available from crop plantations, and they are increasingly used in the pharmaceutical and food industries [2,7].

Analyses of the phytochemical characteristics of the edible and medicinal plants, as well as derived foods, food preparations, and dietary supplements, often focus on polyphenols, yet data on the content of two specific groups of isoprenoid compounds—steroids (including phytosterols) and triterpenoids—are generally lacking. Although present in relatively low quantities, these compounds may significantly contribute to health-supporting benefits. Phytosterols help lower lipid and cholesterol plasma levels, including low-density lipoprotein cholesterol (LDL-C); they are also valued for their antioxidant, anti-inflammatory, hypoglycemic and anticancer properties [16,17,18] and are therefore applied in the prevention of cardiovascular diseases, as well as fatty liver, inflammatory, rheumatoid arthritis, and obesity-related diseases [19,20,21,22]. Triterpenoids, with their structural diversity and a wide range of valuable bioactivities, including anti-inflammatory, antimicrobial, antiviral, hepatoprotective, antidiabetic and anticarcinogenic properties, have wide pharmaceutical and industrial applications [23,24,25]. One of the most promising features of triterpenoids is their potential to be used as novel therapeutic agents against multidrug-resistant microbial and fungal strains [26,27].

The aim of the present study was to analyze the content of these two groups of important phytochemicals in elderberry (*Sambucus nigra*) fruit and commercially available food preparations: jam, juice, syrup, and wine. Although the occurrence of triterpenoids, particularly ursolic acid, in elderberry fruit and flowers has been reported previously [2,3,28], data on the content of other triterpenoids and phytosterols in this plant material remain scarce and warrant further investigation. The content of phytosterols and triterpenoids in elderberry-derived foods and food preparations is largely unexplored. Therefore, the data obtained in this study are particularly valuable, given the increasing popularity of elderberry products due to their widespread promotion as remedies for colds and as immune-boosting preparations.

An additional objective of the study was to compare the content of triterpenoids and phytosterols in fruits and fruit cuticular waxes from wild and cultivated elderberry (c.v. Haschberg), ornamental elderberry (*S. nigra* f. *porphyrophylla* c.v. Black lace “Eva”), and red elderberry (*S. racemosa*). The results of this study may reveal potential chemotaxonomic features within the Sambucus genus.

## 2. Results

### 2.1. Identification of Steroids and Triterpenoids in Extracts Obtained from Fruits and Fruit-Derived Products

The compounds occurring in obtained extracts were analyzed using gas chromatography-mass spectrometry (GC-MS) and identified based on their MS spectra. Steroids and neutral triterpenoids were analyzed without derivatization, while triterpenoid acids were analyzed after methylation (Section 4.6). Identification was further confirmed by comparing the retention time and chromatographic mobility with those of available authentic standards, as well as by referencing MS library data and relevant literature (Table S1).

In all extracts, typical phytosterols were identified, including campesterol [(24R)-ergost-5-en-3β-ol], isofucosterol (24Z-stigmasta-5,24(28)-dien-3β-ol, also known as Δ5-avenasterol], sitosterol [stigmast-5-en-3β-ol] and stigmasterol [(22E)-stigmasta- 5,22-dien-3β-ol], accompanied by oxygenated sterol derivatives (steroid ketones), such as sitostenone (stigmasta-4-en-3-one) and tremulone (stigmasta-3,5-dien- 7-one). The neutral triterpenoids included the monohydroxyalcohols α-amyrin (urs-12-en-3β-ol), β-amyrin (olean-12-en-3β-ol), germanicol (olean-18-en-3β-ol) and taraxasterol (taraxast-20(30)-en-3β-ol), one keton, α-amyrenone, and two aldehydes, oleanolic and ursolic aldehyde.

The main acids identified were ursolic acid (3β-hydroxy-urs-12-en-28-oic acid), oleanolic acid (3β-hydroxy-olean-12-en-28-oic acid) and betulinic acid (3β-hydroxy-lup-20(29)-en-28-oic acid). Additional derivatives of oleanolic and ursolic acids were also detected, including 3-oxo-analogs (3-oxo-olean-12-en-28-oic acid and 3-oxo-urs-12-en-28-oic acid); analogs with an additional double bond at position 2 (olean-2,12-dien-28-oic acid and ursa-2,12-dien-28-oic acid); and a ursane-type acid with an additional hydroxyl group at position 2, corosolic acid (2α,3β-dihydroxy-urs-12-en-28-oic acid). In some extracts, particularly those of ornamental elderberry (*S. nigra* f. *porphyrophylla* c.v. Black lace “Eva”) and red elder (*S. racemosa*), two esters, oleanolic and ursolic acid acetates, were additionally identified. Surprisingly, only trace amounts of oleanolic and ursolic acids were detected in extracts from elderberry syrup and wine. The structures of all the identified triterpenoids are presented in Figures S1 and S2.

### 2.2. The Content of Steroids and Triterpenoids in S. nigra and S. racemosa Fruits

The ornamental black elder *S. nigra* f. *porphyrophylla* cultivar Black lace “Eva” was the richest source of both phytosterols and triterpenoids, with the total content of all identified compounds approaching 1.5% of the fruit dry weight. In contrast, the red elder *S. racemosa* fruit contained only about 0.2% of d.w. of these compounds. Wild elderberry fruit had approximately 33% higher levels of phytosterols and triterpenoids than the cultivated *S. nigra* variety, Haschberg.

Among the phytosterols, sitosterol was the most predominant in all studied fruits, followed by campesterol, which was the second most abundant. Sitosterol represented approx. 75% of the phytosterol fraction in both cultivated and wild elderberries, 57% in the ornamental cultivar Black lace “Eva”, and up to 88% in red elder *S. racemosa*.

The results of the content of individual compounds with reference to the fruit dry weight, expressed as the mean ± SD of three samples, are presented in Table 1.

The most abundant neutral triterpenoid across all fruits was α-amyrin. In cultivated, wild, and ornamental *S. nigra*, the second most abundant neutral triterpenoid was α-amyrenone, whereas, in *S. racemosa*, the second most abundant was ursolic aldehyde, followed by β-amyrin and germanicol, with α-amyrenone being the least abundant.

Triterpenoid acids were the dominant fraction in all analyzed fruits, with the lowest content (0.12% d.w.) in *S. racemosa* fruit; this was followed by 0.41% in the cultivated *S. nigra* cultivar Haschberg and 0.55% in wild *S. nigra*, and the highest content (1.3% d.w.) was detected in ornamental *S. nigra*. Ursolic acid was the most abundant, making up 66–70% of the triterpenoid acid fraction. Notably, *S. racemosa* lacked betulinic and corosolic acids, as well as 3-oxo-analogs of oleanolic and ursolic acids. On the other hand, acetates of oleanolic and ursolic acids were mainly found in *S. racemosa* and ornamental *S. nigra* fruits.

### 2.3. The Content of Steroids and Triterpenoids in Fruit Cuticular Waxes

The general composition of steroids and triterpenoids present in the culticular waxes of the studied fruits was identical to that in the whole fruits (Table 2). However, the proportion of individual groups of compounds, particularly triterpenoid acids, was slightly different. In the fruits of wild elderberry and the cultivated variety Haschberg, triterpenoid acids constituted about 84% of the total content of all identified compounds, in the ornamental elderberry it is 89%, and in the red elder *S. racemosa* only 55% (Table 1). In contrast, in the extracts from cuticular waxes, the percentage content of triterpenoid acids was markedly higher, in wild elderberry and cultivated Haschberg it was 93%, in the ornamental elderberry it was as high as 95%, and, in red elder, we detected 82% of the total content of identified compounds.

### 2.4. The Content of Steroids and Triterpenoids in Elderberry-Derived Food Products

Elderberry jam, syrup, juice, and wine were extracted with diethyl ether, as detailed in Section 4.2; they were then analyzed for their phytosterol and triterpenoid content. The results, reported relative to fresh weight (for jam) or volume (for syrup, juice, and wine), and expressed as the mean ± SD of three samples, are presented in Table 3.

The jam was found to be the richest source of phytosterols and triterpenoids. The results appear to support the producer’s claim of using 35 g of fresh fruit per 100 g of the product. In this case, a 410 g portion of jam would contain approximately 140 mg of phytosterols and triterpenoids (based on a total content of about.4900 µg/g of Haschberg elderberry d.w.), which corresponds to roughly 341 µg per 1 g of jam. This is almost perfectly consistent with the findings of the present study.

Elderberry juice contained fewer phytosterols and triterpenoids than the jam; nevertheless, the content of these compounds can still be considered relatively high for a liquid beverage, given the low polarity of these compounds and their poor solubility in water. The presence of these low-polar compounds is likely attributed to the method of juice production, which, according to the producer, involved 100% natural juice directly pressed from fruit without the addition of water.

The syrup contained approximately three times fewer phytosterols and neutral triterpenoids than juice. However, since only traces of triterpenoid acids were detected during the analysis, the total content of identified compounds in the syrup was five times lower than in the juice. According to the producer, the syrup was made up of 53% juice with added sugar, but no further details regarding the production method were provided. Therefore, it is difficult to explain the relatively large difference in triterpenoid content between the syrup and the juice. Surprisingly, elderberry wine also lacked triterpenoid acids, and the total content of phytosterols and triterpenoids in the wine was the lowest among all the tested products.

## 3. Discussion

### 3.1. Composition of Steroids and Triterpenoids in S. nigra and S. racemosa Fruits

Previous reports [2,29,30,31] identified only a limited number of triterpenoids (amyrins, as well as betulinic, oleanolic and ursolic acids) and two phytosterols (sitosterol and stigmasterol) in *S. nigra* fruits. To our knowledge, the composition of these compounds in the fruits of *S. racemosa* has not yet been determined. Therefore, the present study offers a comprehensive analysis of triterpenoids and phytosterols in the fruits of both black elder (*S. nigra*) and red elder (*S. racemosa*), providing their full profile in fruits and fruit cuticular waxes. The results revealed that the triterpenoid composition of both black elder (cultivated, wild, ornamental) and red elder is complex, comprising compounds with oleanane-, 18-oleanane-, ursane-, lupane- and taraxastane-type carbon skeletons. While the overall triterpenoid composition was similar in both *S. nigra* and *S. racemosa* fruits, there were distinct differences in the quantitative profiles between these species. The most remarkable features distinguishing *S. racemosa* fruit from *S. nigra* include a significantly lower total content of triterpenoids in the whole fruits and fruit cuticular waxes, encompassing both neutral triterpenoids and triterpenoid acids; the abundance of ursolic aldehyde (instead of α-amyrenone, as found in *S. nigra*); and the absence of betulinic and corosolic acids, along with 3-oxo-analogs of oleanolic and ursolic acids.

Interestingly, a comparison of the triterpenoid and steroid profiles of elderberry fruits with those of elderberry inflorescences reported previously [28] revealed notable differences between these two plant organs. For instance, triterpenoid alcohols with an additional hydroxy group, such as erythrodiol (olean-12-ene-3β,28-diol) and uvaol (urs-12-ene-3β,28-diol), were detected in the wild and ornamental elderberry inflorescences, but were absent in the fruits of the same plants. Conversely, betulinic acid was present in the fruits, but not in the inflorescences. Surprisingly, one of the main phytosterols, stigmasterol, was not found in detectable amounts in elderberry inflorescences, whereas it was present in the fruits. These results confirm the widely accepted observation that the content of specific plant metabolites can vary significantly among different plant organs, which may be important for their potential pharmacological applications as sources of bioactive compounds [32,33].

### 3.2. Content of Steroids and Triterpenoids in Fruits and Fruit-Derived Products

The available reports on the content of triterpenoid acids in elderberries typically present values based on the fresh weight of the fruits [2,29,30,31]. The results from this study align with the previous findings when converted to fresh weight. For instance, the ursolic acid content in fresh wild elderberries was previously reported as 734 µg/g f.w. [31], which corresponds to approx. 3670 µg/g d.w. (assuming an 80% hydration level of the fruit). In this study, the ursolic acid content in wild elderberries was estimated as 3595 µg/g fruit d.w., which equals 719 µg/g f.w. Additionally, both studies demonstrate that wild elderberry have a higher level of triterpenoid acids compared to cultivated Haschberg fruits.

As previously mentioned, the elderberry fruit is the most commonly utilized part of the plant; it is widely used to produce a variety of food preparations and dietary supplements [6,13,34,35]. Although elderberries should not be eaten raw, they are considered “superfruits” due to their rich nutrient profile, which includes beneficial compounds such as phenolics (especially anthocyanins), essential oils, carotenoids, polyunsaturated fatty acids, vitamins, and minerals [4,15]. Nevertheless, the processing of fruits into food products can significantly change both the composition and the concentration of some bioactive ingredients. In this regard, the content of triterpenoids and phytosterols in food products is of particular nutritional interest, as, unlike many other bioactive substances, they exhibit notable stability under typical thermal processing conditions, such as boiling temperatures, not being significantly disrupted or altered. Interestingly, in certain vegetables (e.g., beans, carrots, cauliflower, courgettes), the total sterol content may even increase after cooking. This phenomenon is attributed to the hydrolysis of steryl glycosides present in plant tissues, which release free sterols [36]. At higher processing temperatures, such as those encountered during frying in oil, sterols become more prone to oxidation [37]. However, such high-temperature conditions are not typically used in the production of jam, juices, syrups, or wines, where lower thermal treatments prevail. Triterpenoids are generally characterized by their high thermal stability [38]. It was demonstrated that these compounds remained intact during the thermal processing of hawthorn fruits, while other phytochemicals, such as flavonoid glycosides and proanthocyanidins, experienced substantial losses [39].

The occurrence of certain triterpenoids, including oleanolic and ursolic acids, as well as phytosterols such as sitosterol and stigmasterol, have been reported in elderberry fruits as potential health-promoting constituents [2,29,30,31]. However, no data on their presence in elderberry fruit-derived food products have been made available so far. This study represents the first analysis of these compounds in some popular elderberry-based products, i.e., jam, juice, syrup, and wine.

The obtained results revealed that the profile and content of triterpenoids and steroids can vary significantly among different types of food products. As expected, jam was found to be the richest source of phytosterols and triterpenoids, with a total content of approx. 338 µg per 1 g of jam. Thus, elderberry jam can be recommended as a good source of these potentially beneficient constituents. The second most abundant food product was elderberry juice, freshly pressed from the fruits, with a content of approx. 16.38 µg per 1 mL. Syrup and wine contained relatively small amounts of phytosterols and triterpenoids, with only traces of triterpenoid acids. Unfortunately, without knowing the exact method of production, it is difficult to explain these findings.

It is evident that the reported concentrations of the analyzed compounds, e.g., phytosterols, are significantly lower than the recommended therapeutic daily intake, which is estimated to be 1.5 to 3 g per day [40]. To achieve the minimum effective dose required for LDL-cholesterol reduction, one would need to consume more than 4 kg of elderberry jam per day. However, while achieving the therapeutic levels of phytosterols and triterpenoids through diet alone may be challenging, their incorporation into daily diets, especially in combination with other bioactive compounds, can provide a range of health-promoting effects and enhance the bioavailability and efficacy of individual components. Beyond their cholesterol-lowering effect, phytosterols and triterpenoids support cardiovascular health, modulate immune responses, and exert antimicrobial properties [41].

The method of production can be crucial for the triterpenoid content in fruit-derived products due to the presence of these compounds, particularly triterpenoid acids, in the fruit cuticular waxes. Triterpenoid acids are abundant in the cuticular waxes of numerous edible fruits [42], and the present study has shown that *S. nigra* fruits can be added to this list. The obtained results indicate that, in the case of *S. nigra,* the percentage of acids in the total triterpenoid content is higher in the cuticular waxes than in the fruits analyzed as a whole. Therefore, it can be expected that food products that include fruit peel will contain more triterpenoids than those produced only from juice.

Moreover, the origin of the plant material in plant-derived products is often unknown, which is significant, as the content of phytosterols and triterpenoids can differ between wild and cultivated elderberries, as well as various cultivars [31]. Despite the absence of bioactive triterpenoid acids in both the syrup and wine, these food products can still be considered valuable for consumers due to the presence of potentially beneficial anthocyanins and other phenolic compounds [35].

The by-products remaining after food processing (such as fruit pomace generated during wine production) can be used to obtain valuable compounds, such as anthocyanins or triterpenoids [35,43]. Elderberry pomace can serve as a source of food colorants or other functional additives for various food products [44]. Interestingly, the present study revealed that the most abundant source of triterpenoids was ornamental elderberry, which is typically regarded as inedible. Therefore, this fruit could be utilized for the extraction of bioactive triterpenoids, which could, for example, be used in in vitro studies of the bioactivity of these compounds.

## 4. Materials and Methods

### 4.1. Plant Material

**Figure 1:**
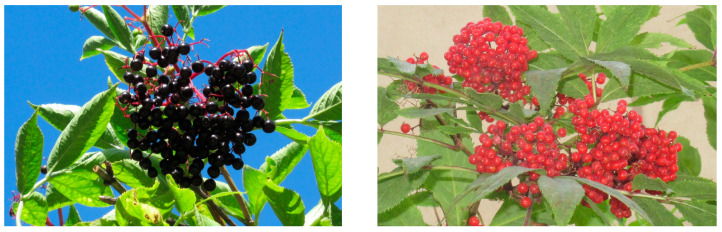
Fruits of wild elderberry *S. nigra* (**left**) and red elder *S. racemosa* (**right**).

Fruits of the cultivated elderberry *Sambucus nigra* cv. Haschberg were purchased from the market at the experimental orchard of the Research Institute of Horticulture in Skierniewice, Poland, in September 2023. Fruits of wild *S. nigra* (Figure 1) and ornamental *S. nigra* f. *porphyrophylla* (c.v. Black lace “Eva”) were collected from a private farm specializing in medicinal and ornamental plants in Stare Bosewo, central Poland (52°460′ N, 21°332′ E) in September 2023. Saplings of these plants were purchased from the licensed supplier of certified crops and ornamental plants, the Polish Vegetable Seed Farming and Nursery enterprise “PNOS”, Ożarów Mazowiecki, Poland, and cultivated in an open field.

The fruits of the red elderberry *S. racemosa* (Figure 1) were collected from an ornamental shrub planted on the Campus Ochota grounds of the University of Warsaw (52°124′ N, 20°505′ E) in July 2023.

### 4.2. Fruit-Derived Products

Elderberry jam (Rapsodia, Agros Nova Food, Krynica, Poland 410 g), syrup (Premium Rosa, Złotokłos, Poland, 250 mL), juice (Oleofarm, Wrocław, Poland, 490 mL), and wine (VIN-KON S.A., Konin, Poland, 750 mL) were purchased from local stores in Warszawa, Poland, in 2024. Triplicates of each product were purchased, with all samples originating from the same producer and the same product batch.

### 4.3. Extraction

The fruits were air-dried at room temperature 25 °C for approximately a week and spread out in a single layer to accelerate drying and prevent mold formation. The dried fruits were weighed and considered dry when they reached a constant weight. Air-dried samples of fruits were powdered in a laboratory mill, weighted, and extracted with diethyl ether (100 mL per sample of approx. 2 g) over the course of 8 h in a Soxhlet apparatus.

The juice, syrup, and jam samples were each extracted three times with diethyl ether using a glass separating funnel (juice and syrup directly, and jam after homogenization in a mixer), applying a 1:2 ratio, i.e., one volume of the organic solvent for two volumes of the extracted sample. To facilitate the separation of the organic phase, alcohol was distilled from the wine prior to the extraction with diethyl ether using a vacuum rotary evaporator. All obtained organic extracts were evaporated to dryness at 40 °C under reduced pressure.

Cuticular waxes were extracted according to the method described earlier [32,33]. Entire undamaged fruits (samples of weigh of approx. 6 g) were extracted via short (30 s) immersion with gentle stirring in the appropriate volume of chloroform (at least 10 times more than the volume of extracted plant material, i.e., approx. 25 mL) at room temperature. All experiments were conducted in three replicates. The obtained extracts were decanted, filtered, evaporated to dryness under a gentle stream of nitrogen, and weighted.

### 4.4. Fractionation of Extracts via Thin-Layer Adsorption Chromatography (TLC)

The 20 cm × 20 cm glass plates were defatted with acetone and manually coated with a 0.25 mm layer of silica gel 60 G (Merck, Darmstadt, Germany). Dried extracts were dissolved in appropriate volumes of diethyl ether and applied linearly onto the silica gel using a glass capillary. Standard solutions of oleanolic acid and sitosterol α-amyrin were applied on the side of the plate parallel to the extract. The plates were developed in chromatographic chambers in the solvent system CHCl_3_/MeOH (97:3, *v*/*v*).

The individual fractions were localized on the plates by comparison with standards and visualized by spraying the relevant part of the plate with 50% H_2_SO_4_, followed by heating with a hot air stream. The gel was divided into two fractions: (i) free (non-esterified) steroids and neutral triterpenoids (alcohols, aldehydes and ketones) and (ii) triterpenoid acids. Fractions were eluted from the gel in diethyl ether using at least 10 volumes of the solvent relative to the volume of the scrapped gel. The fractions containing free neutral triterpenes and steroids (*R*_F_ 0.3–0.9) were analyzed directly via GC-MS, while the fractions containing triterpene acids (*R*_F_ 0.2–0.3) were first methylated with diazomethane.

### 4.5. Methylation of Triterpenoid Acids

Nitrosomethylurea (2.06 g) was added to a mixture of 100 mL of diethyl ether and 6 mL of 25% aqueous KOH. The organic layer was washed with water (3 × 50 mL) and separated from the aqueous layer. Samples containing triterpenoid acids were dissolved in 5 mL of the obtained solution of diazomethane in diethyl ether and held at 2 °C for 24 h.

### 4.6. Identification and Quantification of Steroids and Triterpenoids via Gas Chromatography-Mass Spectrometry (GC-MS)

An Agilent Technologies 7890A gas chromatograph equipped with a 5975C mass spectrometric detector was used for both the qualitative and quantitative analyses. Samples dissolved in diethyl ether:methanol (5:1, *v*/*v*) were applied (in volumes of 1–4 μL) using a 1:10 split injection. All samples were analyzed in triplicate. The column used was a 30 m × 0.25 mm i.d., 0.25-μm, HP-5MS UI (Agilent Technologies, Santa Clara, CA, USA). Helium was used as the carrier gas at a flow rate of 1 mL/min. The separation was carried out using the following temperature program: an initial temperature of 160 °C held for 2 min and then increased to 280 °C at a rate of 5 °C/1 min, and the final temperature of 280 °C was held for an additional 44 min.

The other employed parameters were as follows: inlet and FID (flame ionization detector) temperature 290 °C; MS transfer line temperature 275 °C; quadrupole temperature 150 °C; ion source temperature 230 °C; EI 70 eV; *m*/*z* range 33–500; FID gas (H_2_) flow 30 mL·min^−1^ (hydrogen generator); and air flow 400 mL·min^−1^. Individual compounds were identified by comparing their mass spectra with library data from Wiley 9th ED. and NIST 2008 Lib. SW Version 2010, or previously reported data, as well as by comparison of their retention times and corresponding mass spectra with those of authentic standards, when available. Quantitation was performed using an external standard method based on calibration curves determined for compounds belonging to representative triterpenoid classes: α-amyrin for triterpene alcohols, oleanolic acid methyl ester for triterpene acid methyl esters, and sitosterol for steroids. Chromatograms were processed using Agilent G1701EA GC/MSD Productivity ChemStation software version E.02.01 2010.

### 4.7. Statistical Analysis of Data

Data are presented as the means ± standard deviation of three independent samples analyzed in triplicate. The data were subjected to one-way analysis of variance (ANOVA), and the differences between means were evaluated using Duncan’s multiple-range test. Statistical significance was considered to be obtained at *p* < 0.05.

## 5. Conclusions

The GC-MS analysis of steoids and triterpenoids in black elder *S. nigra* and red elder *S. racemosa* fruits and fruit cuticular waxes revealed the presence of over 20 compounds, including common phytosterols (e.g., sitosterol, campesterol, and stigmasterol) and various neutral and acidic triterpenoids of oleanane, 18-oleanane, ursane, lupane, and taraxastane types. Sitosterol was the dominant phytosterol in all fruits (up to 80% of the phytosterol fraction), α-amyrin was the most prevalent neutral triterpenoid, and ursolic acid was the most abundant triterpenoid acid, representing 66–70% of the fraction. Ornamental black elder (*S. nigra* f. *porphyrophylla* cultivar Black lace “Eva”) had the highest total content of phytosterols and triterpenoids (1.5% of the fruit dry weight), whereas red elder S. racemosa had the lowest content (0.2% d.w.) and lacked several compounds, including betulinic and corosolic aicds. Wild elderberried contained 33% more bioactive compounds than the cultivated Haschberg variety. Cuticular waxes of fruits contined the same compounds as whole fruits but with higher relative levels of tritepenoid acids (up to 95% in ornamental elderberry). This suggests that fruit peel plays a key role in triterpenoid content, which is relevant to food processing. Among fruit-derived products, jam had the highest levels of triterpenoids and phytosterols (approx. 338 µg/g). Juice retained significant levels but syrup and wine had very low content, with triterpenoid acids nearly absent. This variation likely results from processing methods and the absence of peel in some products.

## Figures and Tables

**Table 1:** The content of steroids and triterpenoids in *S. nigra* and *S. racemosa* fruits.

Compound	*S. nigra* Haschberg	*S. nigra* Wild	*S. nigra* f. *porphyrophylla*	*S. racemosa*
	µg/g dry weight			
Phytosterols				
campesterol	75.62 ± 8.25 a	107.58 ± 12.04 b	131.35 ± 12.91 b	40.05 ± 3.90 c
isofucosterol	23.61 ± 2.04 a	29.04 ± 3.16 a	27.83 ± 2.91 a	36.99 ± 4.05 b
sitosterol	410.84 ± 36.18 a	553.68 ± 42.20 b	728.37 ± 68.33 c	664.63 ± 51.19 c
stigmasterol	33.82 ± 3.04 a	49.56 ± 5.18 b	75.97 ± 8.25 c	12.48 ± 1.52 d
sum	543.89	739.86	963.52	754.15
Steroid ketones				
tremulone	19.10 ± 2.06 a	23.79 ± 2.93 a	34.32 ± 3.66 b	25.79 ± 3.90 a
sitostenone	20.97 ± 2.91 a	27.70 ± 3.36 b	53.09 ± 6.75 c	56.85 ± 6.94 c
sum	40.07	51.49	87.41	82.64
Neutral triterpenoids				
α-amyrin	70.85 ± 5.11 a	114.47 ± 20.13 b	173.36 ± 22.80 c	63.70 ± 8.02 a
β-amyrin	39.53 ± 4.87 a	52.20 ± 6.18 b	80.74 ± 9.42 c	24.34 ± 3.88 d
α-amyrenone	52.79 ± 7.4 a	73.58 ± 9.26 b	127.73 ± 15.65 c	9.97 ± 1.53 d
germanicol	14.86 ± 1.64 a	11.71 ± 1.95 a	14.45 ± 1.80 a	20.00 ± 2.96 b
taraxasterol	10.85 ± 1.23 a	9.67 ± 1.51 a	11.82 ± 1.36 a	18.52 ± 2.48 b
oleanolic aldehyde	6.96 ± 0.82 a	10.27 ± 1.03 b	11.77 ± 1.25 b	10.12 ± 1.46 b
ursolic aldehyde	27.41 ± 3.05 a	48.38 ± 5.10 b	44.39 ± 4.67 b	29.96 ± 3.12 a
sum	223.25	320.28	464.26	176.61
Triterpenoid acids				
betulinic acid	36.88 ± 4.56 a	48.12 ± 6.08 b	230.52 ± 25.66 c	n.d.
olean-2,12-dien-28-oic acid	40.13 ± 5.49 a	79.24 ± 9.06 b	92.90 ± 10.35 b	22.12 ± 3.48 c
ursa-2,12-dien-28-oic acid	151.57 ± 12.41 a	202.48 ± 24.06 b	321.20 ± 34.54 c	48.27 ± 5.9 d
3-oxo-oleanolic acid	44.70 ± 5.15 a	53.78 ± 6.02 a	84.83 ± 9.6 b	n.d.
3-oxo-ursolic acid	71.44 ± 8.06 a	109.62 ± 14.56 b	245.09 ± 2.95 c	n.d.
oleanolic acid	778.83 ± 86.65 a	1309.63 ± 154.9 b	2883.61 ± 303.05 c	204.83 ± 25.49 d
ursolic acid	2898.88 ± 314.02 a	3595.10 ± 402.66 b	9245.74 ± 1038.5 c	850.93 ± 98.71 d
corosolic acid	106.40 ± 11.62 a	82.60 ± 9.08 a	110.65 ± 12.7 a	n.d.
oleanolic acid acetate	n.d.	tr.	34.70 ± 4.45 a	28.69 ± 3.44 a
ursolic acid acetate	n.d.	tr.	97.52 ± 10.36 a	81.56 ± 10.64 a
sum	4128.83	5480.57	13,346.76	1236.40
Total	4936.04	6592.20	14,861.95	2249.80

The results are relative to the fruit dry weight and are expressed as the mean ± SD of three independent samples. n.d.—not detected, tr.—traces. Results in rows that do not share a common letter are significantly different (*p* < 0.05).

**Table 2:** The content of steroids and triterpenoids in the cuticular waxes of *S. nigra* and *S. racemosa* fruits.

Compound	*S. nigra* Haschberg	*S. nigra* Wild	*S. nigra* f. *porphyrophylla*	*S. racemosa*
	µg/mg wax extract			
Phytosterols				
campesterol	0.79 ± 0.08 a	1.05 ± 0.11 a	1.22 ± 0.12 a	0.86 ± 0.09 a
isofucosterol	0.23 ± 0.02 a	0.27± 0.02 a	0.25 ± 0.01 a	0.75 ± 0.08 b
sitosterol	7.64 ± 0.62 a	8.97 ± 0.90 a	11.09 ± 1.35 a	7.99 ± 0.51 a
stigmasterol	0.52 ± 0.05 a	0.65 ± 0.06 a	0.98 ± 1.02 b	0.30 ± 0.01 c
sum	9.18	10.94	13.54	9.90
Steroid ketones				
tremulone	3.96 ± 0.42 a	4.82 ± 0.54 a	5.18 ± 0.60 a	2.19 ± 0.11 b
sitostenone	2.81 ± 0.08 a	4.15 ± 0.10 b	6.73± 0.75 c	3.64 ± 0.05 d
sum	6.76	7.97	11.91	4.83
Neutral triterpenoids				
α-amyrin	2.93 ± 0.31 a	3.57 ± 0.41 a	5.32 ± 0.58 b	2.39 ± 0.25 a
β-amyrin	1.05 ± 0.10 a	1.51 ± 0.02 b	2.66 ± 0.30 c	0.96 ± 0.10 a
α-amyrenone	4.08 ± 0.46 a	4.30 ± 0.40 a	4.82 ± 0.44 a	0.31 ± 0.02 b
germanicol	0.93 ± 0.10 a	0.76 ± 0.08 a	0.85 ± 0.10 a	0.88 ± 0.08 a
taraxasterol	2.34 ± 0.24 a	2.25 ± 0.21 a	2.41 ± 0.26 a	0.52 ± 0.06 b
oleanolic aldehyde	1.47 ± 0.11 a	3.31 ± 0.35 b	3.52 ± 0.36 b	0.43± 0.04 c
ursolic aldehyde	5.88 ± 0.62 a	12.01 ± 1.35 b	14.08 ± 1.40 b	1.56 ± 0.14 c
sum	18.68	27.71	33.66	7.05
Triterpenoid acids				
betulinic acid	4.38 ± 0.42 a	5.04 ± 0.52 a	20.95 ± 2.21 b	n.d.
olean-2,12-dien-28-oic acid	4.69 ± 0.48 a	6.98 ± 0.72 b	8.03 ± 0.95 c	1.58 ± 0.11 dc
ursa-2,12-dien-28-oic acid	16.03 ± 1.75 a	20.58 ± 2.46 b	29.78 ± 2.46 c	3.39 ± 0.30
3-oxo-oleanolic acid	4.27 ± 0.43 a	4.72 ± 0.49 a	7.84 ± 8.20 b	n.d.
3-oxo-ursolic acid	8.54 ± 0.90 a	11.69 ± 1.21 b	20.35 ± 2.11 c	n.d.
oleanolic acid	80.14 ± 8.26 a	104.62 ± 12.04 b	249.86 ± 30.14 c	17.36 ± 2.04 d
ursolic acid	298.15 ± 32.40 a	369.55 ± 38.01 b	796.02 ± 82.40 c	70.69 ± 8.05 d
corosolic acid	14.08± 1.46 a	10.12 ± 1.24 b	14.47 ± 1.63 a	n.d.
oleanolic acid acetate	n.d.	tr.	3.15 ± 0.33 a	1.58 ± 0.18 b
ursolic acid acetate	n.d.	tr.	9.58 ± 1.06 a	4.69 ± 0.52 b
sum	430.28	526.32	1160.03	99.29
Total	464.92	572.94	1219.14	121.07

The results are given with reference to the cuticular wax extract mass and are expressed as the mean ± SD of three independent samples. n.d.—not detected, tr.—traces. Results in rows that do not share a common letter are significantly different (*p*< 0.05).

**Table 3:** The content of steroids and triterpenoids in elderberry-derived food products.

Compound	Jam [µg/g]	Juice [µg/mL]	Syrup [µg/mL]	Wine [µg/mL]
Phytosterols				
campesterol	5.36 ± 0.68	1.07 ± 0.15	0.32 ± 0.03	0.070 ± 0.008
isofucosterol	1.64 ± 0.20	0.35 ± 0.04	0.11 ± 0.01	0.003 ± 0.0002
sitosterol	25.28 ± 3.14	5.37 ± 0.65	1.53 ± 0.16	0.510 ± 0.06
stigmasterol	2.36 ± 0.28	0.62 ± 0.07	0.18 ± 0.01	0.030 ± 0.004
sum	34.64	7.41	2.14	0.613
Steroid ketones				
tremulone	1.36 ± 0.12	0.25 ± 0.03	0.10 ± 0.01	0.025 ± 0.003
sitostenone	1.49 ± 0.15	0.30 ± 0.04	0.12 ± 0.01	0.038 ± 0.004
sum	2.35	0.55	0.22	0.063
Neutral triterpenoids				
α-amyrin	3.82 ± 0.40	0.89 ± 0.10	0.20 ± 0.02	0.073 ± 0.008
β-amyrin	2.74 ± 0.32	0.40 ± 0.05	0.09 ± 0.01	0.038 ± 0.004
α-amyrenone	2.52 ± 0.26	0.27 ± 0.03	0.08 ± 0.009	0.030 ± 0.003
germanicol	0.96 ± 0.10	0.12 ± 0.01	0.02 ± 0.003	0.016 ± 0.002
taraxasterol	0.78 ± 0.08	0.10 ± 0.01	0.02 ± 0.002	0.011 ± 0.001
oleanolic aldehyde	0.49 ± 0.05	0.05 ± 0.01	0.01 ± 0.001	0.006 ± 0.0008
ursolic aldehyde	1.86 ± 0.20	0.16 ± 0.02	0.04 ± 0.005	0.021 ± 0.003
sum	13.17	1.99	0.82	0.195
Triterpenoid acids				
betulinic acid	n.d.	0.06 ± 0.007	n.d.	n.d.
olean-2,12-dien-28-oic acid	2.86 ± 0.34	0.11 ± 0.02	n.d.	n.d.
ursa-2,12-dien-28-oic acid	10.83 ± 1.46	0.42 ± 0.05	n.d.	n.d.
3-oxo-oleanolic acid	3.19 ± 0.45	0.05 ± 0.006	n.d.	n.d.
3-oxo-ursolic acid	5.08 ± 0.58	0.09 ± 0.010	n.d.	n.d.
oleanolic acid	57.63 ± 6.81	1.36 ± 0.18	tr.	tr.
ursolic acid	202.43 ± 25.03	4.11 ± 0.53	tr.	tr.
corosolic acid	6.57 ± 0.87	0.01 ± 0.001	n.d.	n.d.
oleanolic acid acetate	n.d.	0.07 ± 0.008	n.d.	tr.
ursolic acid acetate	n.d.	0.15 ± 0.02	n.d.	tr.
sum	288.59	6.43	0	0
Total	338.75	16.38	3.18	0.871

Results are given with reference to the mass or volume of fruit-derived product and are expressed as the mean ± SD of three samples. n.d.—not detected, tr.—traces.

## Data Availability

Data are contained within the article and supplementary materials.

## Supplementary Materials

The following supporting information can be downloaded at: https://www.mdpi.com/article/10.3390/plants14101490/s1: Figure S1. The structures of steroids and neutral triterpenoids identified in extracts from elderberry fruits. Figure S2. The structures of triterpenoid acids identified in extracts from elderberry fruits. Table S1: Retention times and characteristic ions of the mass spectra of identified steroids and triterpenoids.

## Abbreviations

The following abbreviations are used in this manuscript:

COVID-19	Coronavirus disease of 2019
FID	Flame ionization detector
GC-MS	Gas chromatography coupled with mass spectrometry
IL-6	Interleukin 6
IL-8	Interleukin-8
LDL-C	Low-density lipoprotein cholesterol
MERS	Middle East respiratory syndrome
N.d.	Not detected
SARS	Severe acute respiratory syndrome
S.D.	Standard deviation
TLC	Thin-layer chromatography
TNF	Tumor necrosis factor
Tr.	Traces
