# Supplementary material for: Fruit and Fruit-Derived Products of Selected Sambucus Plants as a Source of Phytosterols and Triterpenoids
Source: Plants (Basel). 2025 May 16;14(10):1490. doi: 10.3390/plants14101490 (PMC12114898; doi:10.3390/plants14101490)
Supplement: Supplementary file 1 [file plants-14-01490-s001.zip › plants-3604775-supplementary.pdf]

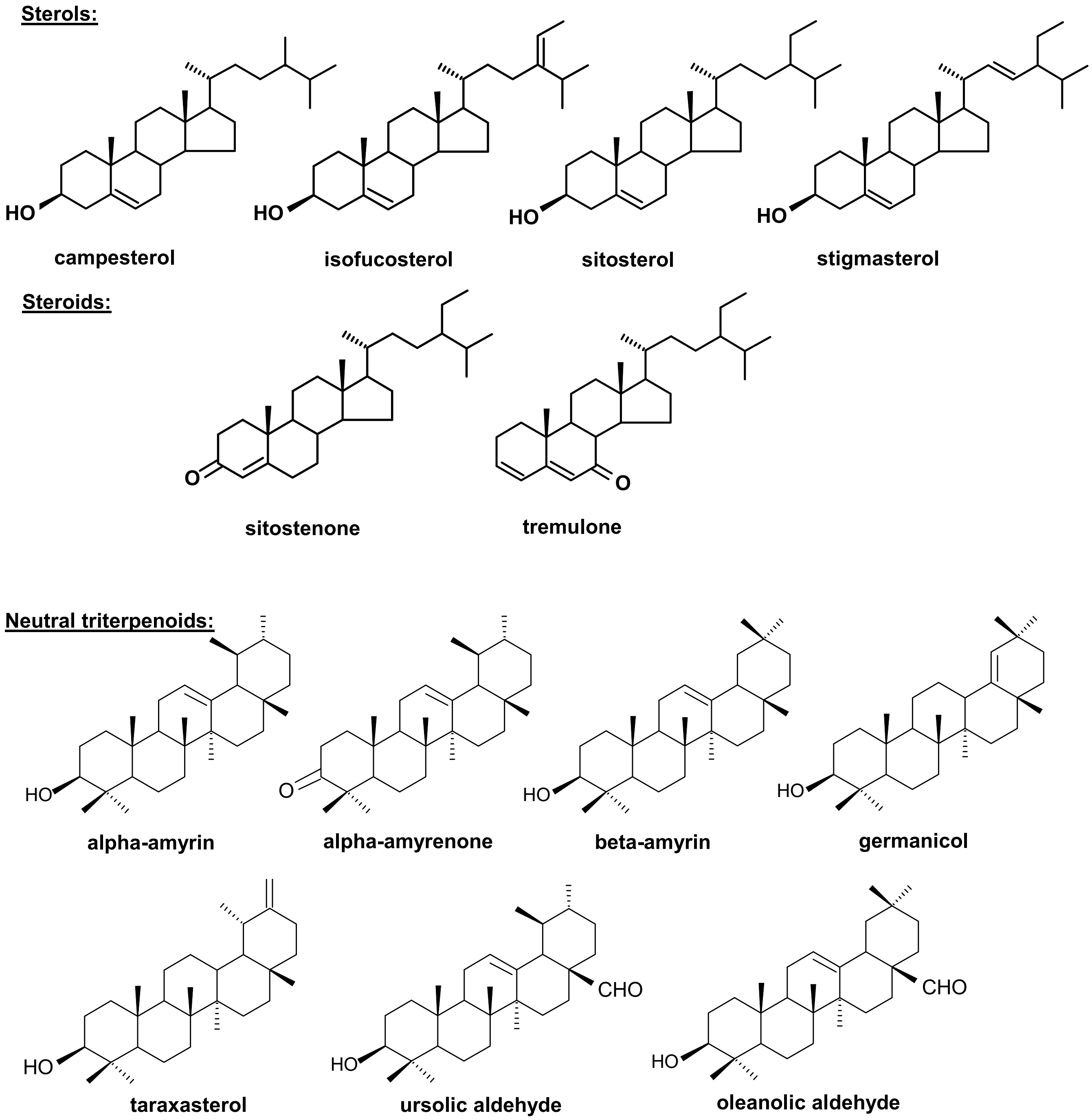

Figure S1. The structures of steroids and neutral triterpenoids identified in extracts from elderberry fruits.

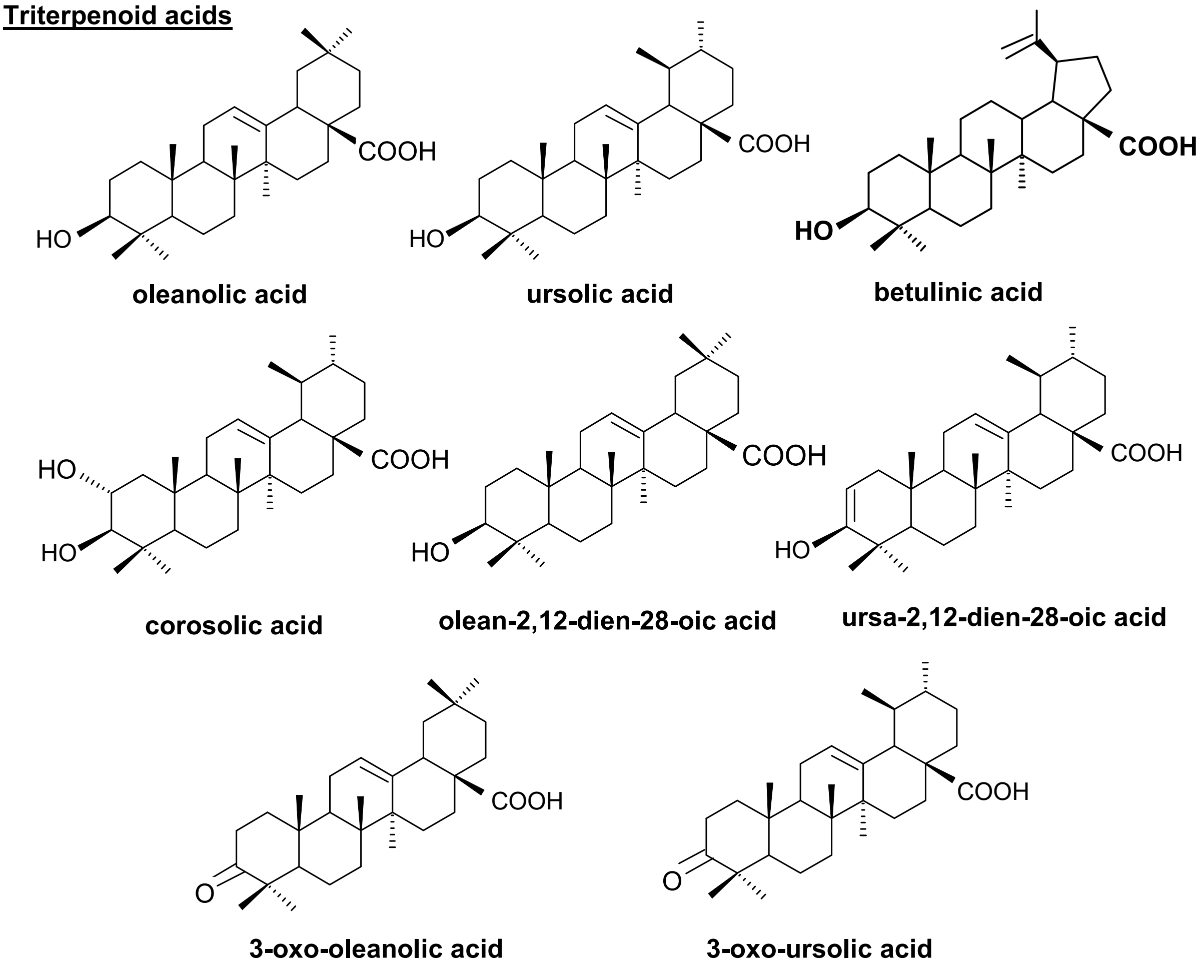

Figure S2. The structures of triterpenoid acids identified in extracts from elderberry fruits.

Table S1. Retention times and characteristic ions of mass spectra of identified steroids and triterpenoids

Retention time (min)	Compound	Mass spectrum m/z (relative intensity)
34.2	Campesterol*	400 (30), 107 (51), 105 (55), 95 (49), 83 (45), 81 (64), 71 (62), 57 (77), 55 (77), 43 (100), 41 (52)
35.1	Stigmasterol*	412 (36), 145 (64), 107 (52), 95 (100), 83 (66), 81 (90), 78 (60), 69 (67), 67 (85), 55 (69)
36.9	Sitosterol*	414 (29), 145 (54), 107 (59), 105 (60), 95 (54), 91 (49), 81 (57), 57 (68), 55 (70), 43 (100), 41 (44)
37.4	Isofucosterol*	412 (5), 314 (100), 105 (47), 95 (50), 91 (42), 83 (40), 81 (51), 69 (61), 55 (96), 43 (49)
37.8	Germanicol	426 (1), 204 (100), 177 (85), 189 (75), 95 (58), 55 (46), 205 (44), 81 (42), 109 (40), 69 (39), 107 (37)
38.1	β-Amyrin*	426 (27), 219 (18), 218 (100), 203 (49), 189 (17), 135 (11), 109 (13), 105 (12), 95 (15), 81 (18), 69 (14)
38.6	α-Amyrenone*	424 (12), 219 (19), 218 (100), 203 (24), 189 (16), 135 (19), 133 (18), 122 (18), 119 (17), 95 (16), 55 (18)
39.2	α-Amyrin*	426 (4), 218 (100), 203 (20), 189 (36), 135 (35), 121 (32), 109 (32), 107 34), 95 (40), 81 (33), 55 (31)
39.7	Tremulone (stigmasta-3,5-dien-7-one)	410 (32), 187 (27), 174 (100), 161 (37), 159 (26), 91 (28), 57 (28), 55 (37), 43 (44), 41 (28)
41.2	Sitostenone	412 (37), 229 (34), 218 (31), 124 (100), 109 (31), 95 (41), 81 (27), 69 (32), 55 (37), 43 (44)
43.5	Taraxasterol*	426 (14), 207 (57), 189 (100), 135 (51), 121 (74), 109 (57), 107 (62), 95 (70), 93 (47), 81 (48), 67 (43)
47.8	Oleanolic aldehyde	440 (2), 232 (28), 207 (20), 204 (39), 203 (100), 189 (29), 105 (18), 81 (19), 69 (20), 55 (29)
51.6	Ursolic aldehyde	440 (1), 207 (26), 204 (23), 203 (100), 133 (42), 119 (18), 105 (18), 95 (18), 81 (18), 55 (18), 43 (20)
Acids (analyzed after methylation):		
41.9	Olean-2,12-dien-28-oic acid methyl ester	452(11), 425 (9), 263 (11), 262 (61), 221 (14), 203 (100), 190 (15), 189 (22), 133 (14), 119 (12)
44.7	3-Oxo-olean-12-en-28-oic acid methyl ester*	468 (6), 262 (32), 204 (17), 203 (100), 202 (21), 189 (29),133 (17), 119 (14), 105 (12), 55 (12)
46.6	Oleanolic acid methyl ester	470 (1), 262 (48), 207 (13), 204 (16), 203 (100), 202 (21), 189 (22), 133 (17), 119 (13), 105 (14)
47.0	Betulinic acid methyl ester*	470 (5), 207 (41), 203 (38), 189 (100), 175 (40), 119 (41), 107 (38), 105 (37), 95 (37), 93 (38)

47.2	3-Oxo-urs-12-en-28-oic acid methyl ester*	468 (3), 263 (21), 262 (96), 249 (20), 204 (17), 203 (100), 189 (29), 133 (79), 119 (30), 105 (19)
49.7	Ursolic acid methyl ester*	470 (1), 263 (20), 262 (100), 207 (32), 203 (93), 189 (29), 133 (76), 119 (34), 105 (21), 95 (18)
63.0	Corosolic acid methyl ester*	486 (1), 263 (15), 262 (74), 204 (17), 203 (100), 202 (22), 189 (21), 119 (18), 105 (14), 55 (12)

*identified by the comparison with authentic standard
